# Antibody and T-Cell Responses against SARS-CoV-2 after Booster Vaccination in Patients on Dialysis: A Prospective Observational Study

**DOI:** 10.3390/vaccines11020260

**Published:** 2023-01-25

**Authors:** Moe Kawashima, Hiroaki Saito, Takamitsu Nishiuchi, Hiroki Yoshimura, Masatoshi Wakui, Yuta Tani, Yoshitaka Nishikawa, Fumiya Omata, Morihito Takita, Tianchen Zhao, Chika Yamamoto, Yurie Kobashi, Takeshi Kawamura, Akira Sugiyama, Aya Nakayama, Yudai Kaneko, Toyoaki Sawano, Kenji Shibuya, Junichiro Kazama, Ryuzaburo Shineha, Masaharu Tsubokura

**Affiliations:** 1 Department of Radiation Health Management, Fukushima Medical University School of Medicine, Fukushima 960-1247, Japan; 2 Soma Central Hospital, Fukushima 976-0016, Japan; 3 School of Medicine, Hiroshima University, Hiroshima 739-8511, Japan; 4 Department of Laboratory Medicine, Keio University School of Medicine, Tokyo 108-8345, Japan; 5 Medical Governance Research Institute, Tokyo 1080074, Japan; 6 Department of General Internal Medicine, Hirata Central Hospital, Fukushima 963-8202, Japan; 7 Research Center for Advanced Science and Technology, The University of Tokyo, Tokyo 153-8904, Japan; 8 Isotope Science Center, The University of Tokyo, Tokyo 113-0032, Japan; 9 Medical & Biological Laboratories Co., Ltd., Tokyo 105-0012, Japan; 10 Department of Surgery, Jyoban Hospital of Tokiwa Foundation, Fukushima 972-8322, Japan; 11 Tokyo Foundation for Policy Research, Tokyo 106-6234, Japan; 12 Department of Nephrology, Fukushima Medical University School of Medicine, Fukushima 960-1247, Japan

**Keywords:** COVID-19 vaccines, renal dialysis, humoral immunity, cellular immunity

## Abstract

Intensive vaccination is recommended for populations more vulnerable to COVID-19 infection, although data regarding the built of immunity after vaccination for dialysis patients are lacking. This prospective, observational cohort study of maintenance hemodialysis patients examined IgG antibody levels against the SARS-CoV-2 spike (S1) protein, neutralizing activity, and interferon gamma levels after the third dose of the BNT162b2 (Pfizer–BioNTech) or mRNA-1273 (Moderna) vaccine. Humoral immunity was repeatedly measured for up to two months. The study includes 58 patients on hemodialysis. Median neutralizing antibodies reached a maximum at 56 and 9 days after booster vaccination with BNT162b2 and mRNA-1273, respectively. The median IgG antibody titer reached a maximum of 3104.38 and 7209.13 AU/mL after 16 days of booster dose, and cellular immunity was positive in 61.9% and 100% of patients with BNT162b2 and mRNA-1273 vaccination, respectively. By repeating the measurements over a period of two months, we clarified the chronological aspects of the acquisition of humoral immunity in dialysis patients after a booster COVID-19 vaccination; most dialysis patients acquired not only humoral immunity, but also cellular immunity against SARS-CoV-2. Future research should investigate the continued long-term dynamics of antibody titers and cellular immunity after the third or further vaccinations, evaluating the need for additional vaccinations for hemodialysis patients.

## 1. Introduction

Severe acute respiratory syndrome coronavirus 2 (SARS-CoV-2), the virus that causes the disease known as COVID-19, has posed a significant global health threat since its emergence in early 2020. In elderly and immunocompromised patients, the severity and fatality rates of COVID-19 disease are particularly high [1,2]. Patients receiving dialysis are also a priority group for the control of COVID-19 infection. Globally, 3.9 million patients are on dialysis worldwide, and the number is increasing [3]. Owing to immune disfunction, patients with an end-stage renal disease requiring dialysis have an increased susceptibility to general infection, which is the second most common cause of mortality worldwide [4]. The mortality rate of COVID-19 infection among dialysis patients (9.0%) is higher than that of the general population (0.3%) [5]. Therefore, patients on dialysis are recommended to take precautions against COVID-19 infection, and measures to prevent dialysis patients from developing serious illness or death from COVID-19 infection are critical.

Vaccines against COVID-19 infection have been developed and administered to prevent the rapid spread of COVID-19, with priority given to medically vulnerable individuals. However, the immune response to COVID-19 induced by vaccination in patients on dialysis has been reported to be poor and short-lived [6,7]. According to Davidovic et al., seroconversion rates and anti-SARS-CoV-2 spike immunoglobulin G (IgG) levels significantly decreased between 4 weeks and 6 months after the second dose of vaccination [8]. Similarly, SARS-CoV-2-specific T-cell responses after the second dose of vaccination were lower in patients on dialysis (52.6%) than in those in the general population (75.0%) [9]. Therefore, the need for a third booster dose has been indicated for patients receiving hemodialysis. Prior studies found that the third dose of the vaccine reduced mortality and hospitalization rates [10]. However, few studies have examined the persistence and establishment of immunity after the third vaccination in hemodialysis patients, and the evolution of antibody titers and the state of T-cell immunity after the third vaccination requires detailed investigation.

Vaccination has been recommended in many countries, and a total of 12.16 billion doses have been administered, with 4.02 million doses still being administered daily as of 13 July 2022 [11]. In Japan, administration of the third dose of vaccinations for medically vulnerable people including patients on dialysis and healthcare workers began on 1 December 2021, 10 months after the first dose in February 2021. By August 2022, 76.5% of the population in Japan had received the second dose of the vaccine and 64.3% had received the third dose [12]. To explore the relationship between the dynamics of post-vaccine immunity and individual factors, lifestyle, and response to vaccines, we established large vaccine cohorts in rural areas of Fukushima prefecture, Japan (Fukushima vaccination cohort) [13,14,15]. In the cohort study, a significant decrease in anti-SARS-CoV-2 S1 and neutralizing antibodies was observed in hemodialysis patients compared to a matched cohort after the second dose of vaccination [16]. The current study further focused on hemodialysis patients and followed them after the third vaccination. We measured the antibody titers before and after booster vaccination, and cases with high frequency of measurement were followed up a total of 14 times over 2 months. In addition, we measured the cellular immunity after booster doses in the same group. This study aimed to analyze the longitudinal changes in antibody titers, reveal the relationship between cellular immunity and antibody titers, and identify the factors associated with the increase in antibody titer among patients on dialysis after the booster COVID-19 vaccine. The chronological evaluation of humoral immunity after the third vaccination in hemodialysis patients, as well as the establishment of cellular immunity, will contribute to the elucidation of the post-vaccine immunity building process and guide the discussion on the need for additional doses of COVID-19 vaccine for hemodialysis patients in the future.

## 2. Materials and Methods

### 2.1. Study Design and Population

In this prospective observational study, all the patients on maintenance dialysis in Soma Central Hospital, Fukushima, Japan, who had completed the third dose of BNT162b2 (Pfizer–BioNTech) vaccine or mRNA-1273 (Moderna) and had no history of COVID-19 infection were eligible for inclusion. The maintenance dialysis patients at this hospital have consistently been on dialysis for years. The information on medical history, comorbidities, and major medications was obtained from the hospital records and questionnaires from the patients. In the questionnaire, optional answers regarding adverse reactions after vaccination were sought (Supplementary file). Blood samples were taken at the time of third vaccination and at the time of each dialysis thereafter. Those who were unable to give a blood sample at the time of the third vaccination or after the vaccination or who did not agree to participate in the study were excluded from the study. All participants were asked to fill out questionnaires regarding the participant details including age, sex, dates of COVID-19 vaccination, adverse reactions after vaccination, prescribed medication, and history of disease. Participation in the study was voluntary. The Public Health Office of Soma City (Fukushima, Japan) broadly informed the residents of this study and written informed consent was obtained prior to recruitment. The hospital staff and municipal employees assisted the elderly participants who could not fill out the paper questionnaires, and informed consent was obtained from the family members of those who could not sufficiently understand the study procedure.

### 2.2. Outcome Measures 

All blood samples were collected between 21 September 2021, and 28 March 2022. The blood samples were centrifuged at each facility and the serum samples obtained were delivered to Tokyo University. All serological assays were performed at Tokyo University between 22 September 2021, and 27 May 2022. Levels of immunoglobulin G (IgG) antibody against the SARS-CoV-2 spike (S1) protein and neutralizing activity were measured as the primary outcome of the immune status after the second and third doses of vaccination. IgG antibody titers against the SARS-CoV-2 nucleocapsid (N)-protein were used to determine previous COVID-19 infection. In addition, cellular immunity was assessed by measuring the number of T-cells stimulated by specific antigens using the T-SPOT^®^.

### 2.3. Serological Assay

The titers of anti-SARS-CoV-2 (wild type) S1- and N1- protein IgG as well as the neutralizing activity were measured using iFlash 3000 (YHLO Biotech, Shenzhen, China) and iFlash-2019-nCoV series assay kits (YHLO Biotech, Shenzhen, China). Measurements were performed in accordance with the manufacturer’s instructions. The validation process for quality control was performed daily before measurement. The cut-off titers of anti-S1 and N1 antibodies, and neutralizing activity were 10 arbitrary units per mL (AU/mL). 

### 2.4. T Cell Response Measurement

In all cases, 10 mL of peripheral blood in lithium heparin and EDTA tubes was obtained from all patients. T-cell responses were assessed using an ELISPOT-based IFN-γ release assay (T-SPOT^®^) utilizing peptide pools derived from the SARS-CoV-2 spike and nucleocapsid proteins. Peripheral blood mononuclear cells (PBMCs) were isolated from whole blood and 250,000 ± 50,000 PBMCs were added per well. The COVID assay (Oxford Immunotec, Abingdon, UK), an ELISPOT assay, was modified to measure SARS-CoV-2-specific T-cell responses. Each well contained an optimized antigen pool containing the SARS-CoV-2 structural protein to stimulate T-cells in vitro and induce IFN-γ production. The IFN-γ released from the cells was captured by antibodies coated at the bottom of the wells. Following incubation for 16–20 h, AP-conjugated secondary antibodies were added to bind to IFN-γ in the solid phase. Subsequently, substrates against the AP were added and the reaction displayed IFN-γ-producing spots. Along with the negative and positive controls, SARS-CoV-2 spike antigen, and SARS-CoV-2 nucleocapsid antigen, a total of four wells were used for each patient’s sample. The peptides were 15-mer peptides with 11 overlapping amino acids, and the pool consisted of 253 peptides for antigen stimulation. The peptides were designed to be presented by MHC class I or class II molecules, the former activating CD8+ T-cells and the latter activating CD4+ T-cells. The results were interpreted by counting the spots in each well and subtracting the number of spots in the negative control as the background from the number of spots in the wells stimulated with the antigen. If the number of spots in the negative control was >10, the test was considered invalid. Following the manufacturer’s recommendations and criteria for spot-counting, four or fewer spots were considered non-reactive, 5–7 were borderline, and >8 were considered reactive.

### 2.5. Statistical Analysis

Data on neutralizing antibodies (Nab) and IgG levels from the day of the third vaccination to March 29 (i.e., 61 days after BNT162b2 vaccination and 40 days after mRNA-1273 vaccination) were used. Multivariable logistic regression analysis was also performed to determine the relationship between anti-S protein specific IgG (IgG (S)) and IFN-γ forming spots. The dependent variable was the number of spots obtained from the T-Spot test, which was divided into two groups: less than 7 or greater than 8. The independent variables included IgG levels against the S protein, sex, age, vaccine type, presence of systemic adverse reactions (fever (≥37.5 °C), malaise, headache, joint pain, and myalgia), and presence of local adverse reactions (pain, swelling, redness at the vaccination site, and fever (<37.5 °C)). In addition, we analyzed factors associated with the highest IgG(S) levels. Each item in the questionnaire was analyzed using linear regression analysis to reveal the relationship with the antibody titer. Multiple linear regression analysis was performed using the items reported to be relevant in previous reports: age, sex, vaccine type, and adverse reactions (local and systemic reactions). All missing values in the data were assigned using the multiple imputation method, and the least-squares error was used for the error function of the model. Statistical significance was defined as a *p*-value ≤ 0.05. All analyses were performed using Python version 3.7, and Google Colaboratory was used as the experimental environment.

## 3. Results

### 3.1. Participant Characteristics

Of the 68 patients undergoing dialysis in Soma Central hospital, 58 were eligible for the study. Of the 10 excluded patients, 5 did not have their blood taken on the day of the third vaccination, 4 never had their blood drawn after the third vaccination, and 1 did not receive the third vaccination. The patients received the first dose of the vaccine between 3 May and 21 June 2021, and the second dose between 3 June and 13 July 2021. They received the third dose between 26 January and 17 February 2022. The first and second vaccines were BNT162b2, whereas 43 patients received BNT162b2 and 15 patients received mRNA-1273 for the third dose. The mean duration between the first and second vaccines was 22 days, and the mean duration between the second and third vaccines was 233 days.

Table 1 summarizes the patient characteristics. Of the 58 patients, 18 (31.0%) were females. The median patient age was 71 years (range: 48–89 years). All the patients underwent standard hemodialysis. The most common medications used by the patients were antihistamines (13.8%), followed by acetaminophen (6.9%). Joint pain was the most common side effect reported after both the second and third doses (34.5% and 70.7%, respectively).

None of the patients were on biological therapeutics among the medications used. Dizziness and diarrhea were among the adverse reactions reported post the third vaccination.

### 3.2. SARS-CoV-2 Antibody Titer

**Figure 1:**
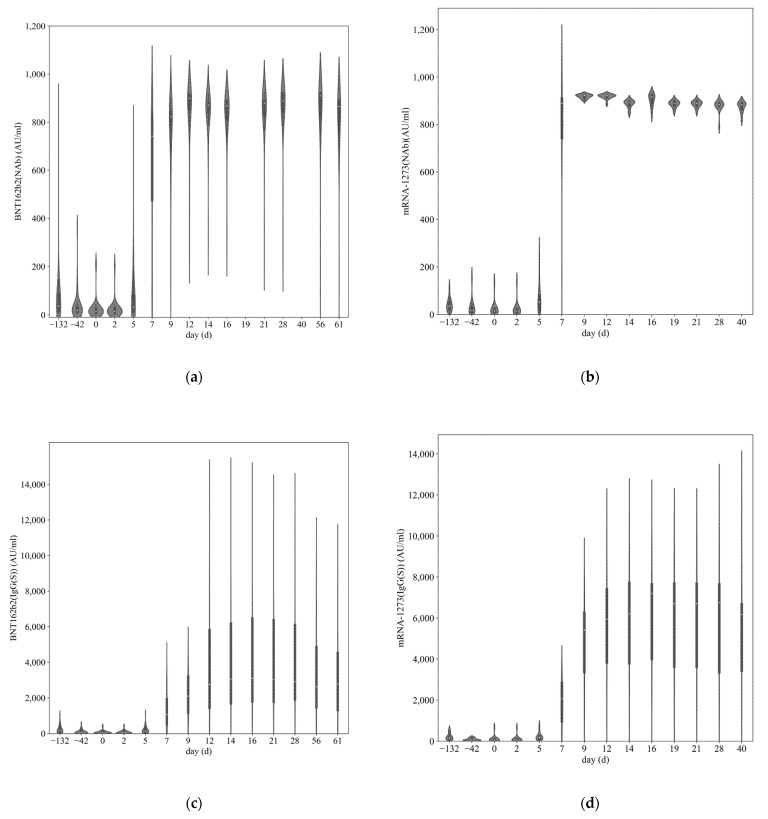
Transition of neutralizing antibody and immunoglobulin G antibody against the SARS-CoV-2 spike protein among dialysis patients. The horizontal axis represents the number of days since the third vaccination date, where day 0 is the third vaccination date and the minus sign represents the number of days before the third vaccination date. Violin plots show the levels of neutralizing activity before and after BNT162b2 (**a**) and mRNA-1273 (**b**) vaccination, as well as IgG levels before and after BNT162b2 (**c**) and mRNA-1273 (**d**) vaccination (AU/mL). The median value is depicted as a point inside each violin plot; the thick bar in the center represents the interquartile range; the thin line represents the upper/lower adjacent value; the width of the density plot shows the frequency of the value.

None of the subjects had SARS-CoV-2 N-IgG antibodies above the cut-off titers (10 AU/mL) throughout the study. The violin plot represents Nab and SARS-CoV-2 IgG (S) antibody titers based on the vaccine type, BNT162b2, and mRNA-1273 (Figure 1a–d). SARS-CoV-2 Nab and S1-IgG antibody titers increased after both BNT162b2 and mRNA-1273 vaccination and rose sharply on day 7. The median BNT162b2 Nab levels reached a maximum of 909.41 [IQR: 822.58–919.86] AU/mL on day 56 and mRNA-1273 Nab levels reached a maximum of 922.25 [IQR: 913.95–924.93] AU/mL on day 9. The median BNT162b2 S1-IgG antibody titer reached a maximum of 3104.38 [IQR: 1798.94–6467.67] AU/mL on day 16, and the median mRNA-1273 S1-IgG antibody titer reached a maximum of 7209.13 [IQR: 3725.13–7731.16] AU/mL on day 16.

### 3.3. Association of IgG(S) Antibody Titers with T-SPOT

**Figure 2:**
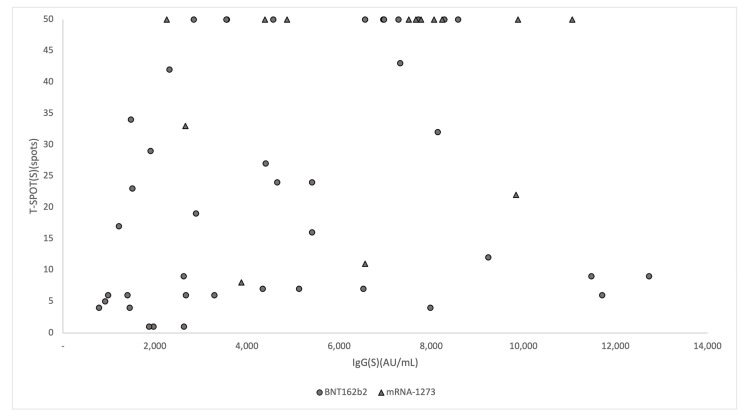
Scatter-plot of the level of IgG antibodies and the number of spots in T-SPOT. The circles represent the values for those who received the third dose of BNT162b2 and the triangles represent the values for those who received the third dose of mRNA-1273.

Blood samples for the T-Spot test were collected on day 56 after vaccination with BNT162b2 and on day 40 after vaccination with mRNA-1273. Blood sample for one patient vaccinated with mRNA-1273 could not be obtained resulting in a total number of 56 patients. 26 (61.9%) of those vaccinated with BNT162b2 and 14 (100%) of those who received the mRNA-1273 vaccine were positive for the S antigen. The correlation between T-SPOT and IgG (S) antibody titers on the same day is shown in Figure 2. Since all patients who received additional vaccination with mRNA-1273 were positive for T-SPOT, multivariable logistic regression analysis was performed on those who received BNT162b2 for the third dose of vaccination. Logistic regression analysis showed that age (adjusted odds ratio [aOR] 0.90, 95% CI 0.82–0.99, *p* = 0.04) was significantly associated with a positive T-SPOT result, while IgG (aOR 1.00 95% CI 1.00–1.00, *p* = 0.12), female sex (aOR 1.52, 95% CI 0.28–8.38, *p* = 0.63), presence of systemic side effects (aOR 0.22, 95% CI 0.29–1.63, *p* = 0.14), and presence of local side effects (aOR 0.82, 95% CI 0.09–7.60, *p* = 0.06) were not significantly associated. 

### 3.4. Factors Associated with Maximum IgG(S) Antibody Titer

In the linear regression analysis, age (coefficient = −81.8 (−159–4.99), *p* = 0.04), inoculation site reaction (coefficient = 3025 (828–5223), *p* < 0.01), and fever (≥37.5 °C) (coefficient = 2505 (519–4492), *p* = 0.01) were associated with the maximum antibody titer after the third vaccination. (Table 2) A multiple regression analysis using age, sex, type of the third vaccine, presence of systemic side effects, and presence of local side effects revealed no statistically significant association with the maximum antibody titers.

Systemic adverse reactions include fever (≥37.5 °C), malaise, headache, joint pain, and myalgia. Local adverse reactions included inoculation site reaction and fever (<37.5 °C).

## 4. Discussion

We analyzed the SARS-CoV-2 antibody titer and T-cell responses in patients on dialysis after the third dose of SARS-CoV-2 vaccination. The antibody titers were followed up in detail over a period of approximately two months to determine changes in the titers and to elucidate the mechanism of increase in antibody titers following vaccination. In this study, antibody titers reached the maximum values at 2 and 3 weeks, and cellular immunity was positive in 61.9% of BNT162b2-vaccinated and 100% of mRNA-1273-vaccinated participants. 

Our study showed that patients on hemodialysis could acquire antibody titers one to two weeks after the third vaccination with the highest value similar to that previously reported [17]. Studies of IgG antibody titers among patients on dialysis showed that antibody titers were higher after the third vaccination than after the second vaccination [18,19]. However, maximum IgG antibody titers may be lower in patients on dialysis than those in the general population. Fucci et al. Reported that in patients on dialysis, antibody titers one month after the third dose of vaccination were 50 times higher than those after the second dose of vaccination, with a median IgG titer of 2232 ng/mL [17], which was approximately one-tenth of the maximum IgG titer of the general population [20]. Consistent with this study, our study showed the highest median IgG titer of 3104.38 AU/mL (boosted by BNT162b2) and 7209.13 AU/mL (boosted by mRNA-1273). Thus, compared with the healthy population, the booster effect of the third vaccine may not be as strong in patients on dialysis. Although our analysis did not reveal any factors related to antibody titers, previous studies have revealed that sex and age affect antibody titers, and other factors such as immunosuppressive status, blood pressure, heart failure, C-reactive protein levels, antibody titers after the second vaccination, and the presence of active cancer are also associated with antibody titers [7,17,21]. The limited number of participants in the present study restricted the examination of factors related to antibody titers, and further research is required. Previous studies have also shown that heterologous boosters increase neutralizing titers in the general population compared to the homologous boosters [22]. Similarly, in this study, all patients on dialysis received the BNT162b2 vaccine for the first and second doses; those who received mRNA-1273 for the third dose showed higher antibody titers than those who received the same vaccine (BNT162b2) for the third dose, although the difference was not significant. In this regard, the type of vaccine may influence the antibody titers in patients on dialysis.

Our study also highlights the importance of considering individual differences in the acquisition of humoral immunity. We found that both IgG and neutralizing antibody titers in some patients did not increase significantly after the third vaccination. Antibody titers in these patients exhibited a sluggish increase, with modest maximum levels, and a steady decline. Regarding the efficiency of vaccinations in preventing severe COVID-19 infection in these participants, a deeper understanding of the effect of neutralizing activity and T-cell responses in protection against infection is necessary. Even after the third vaccination dose, these patients may be susceptible to COVID-19, and it is necessary to identify factors that reduce the antibody titers.

In this study, we analyzed the shifts in antibody titers at short intervals after the third vaccination. Few studies have measured antibody titers every few days after vaccination, which may provide useful information regarding the establishment of humoral immunity. For the first and second vaccination for SARS-CoV-2, previous studies have shown that antibody titers increase from 1 week to 1 month after vaccination in the general population, reach their highest values at 1 month, and gradually decrease afterwards [23,24,25]. Ann et al. found that the neutralization titers against wild-type SARS-CoV-2 in the general population similarly reached sufficient values a week after the third dose of vaccination with BNT162b2 [26]. The results of their study are comparable to those of our study, suggesting that the timeline for building humoral immunity after a third dose of vaccination in patients receiving dialysis is similar to that in the general population. Future research should investigate the continued long-term dynamics of antibody titers in individuals after the third vaccine dose, as well as the factors contributing to reduced antibody titers while evaluating the need for additional vaccinations in patients on hemodialysis.

Previous studies have shown that cellular immunity is lower in older people and those with underlying diseases [27]. In patients on dialysis, the rate of acquiring T-cell immunity within one month of the second vaccination is consistently lower than that in the general population (89–96%), although a wide range of results has been obtained, ranging from 58% to 92% [24,25,26,27]. In terms of the third vaccine type, a better cellular response was reported in patients who received mRNA-1273 than in those who received a BNT162b2 vaccination [28]. The present results are similar to the previously reported results after the second vaccination for patients on dialysis, with different rates of T-cell immunity suggested between BNT162b2 (61.9%) and mRNA-1273 (100%). The 100% positivity rate of cellular immunity following mRNA-1273 vaccination in the current study, conducted with a small number of participants, requires careful consideration regarding the impact of different vaccine types on the acquisition of cellular immunity after the third dose in patients on dialysis. In addition, in this study, we measured the association between cellular and humoral immunity; however, no obvious relationships were observed. Similarly, no clear correlation between antibody titer and cellular immunity against SARS-CoV-2 after vaccination was observed in a previous study [29]. Furthermore, few studies have examined the relationship between cellular and humoral immunity after the third immunization, particularly in patients undergoing hemodialysis; therefore, further research is required. It has been suggested that cellular immunity acquired after the vaccination may complement humoral immunity and clinically protect the vaccinated individuals. Although many viral variants of concern (VOCs) can evade humoral immunity, vaccine-induced cellular responses exhibit strong cross-protection against VOCs, and cellular immunity is said to contribute to disease control [30]. Therefore, it is important to measure cellular immunity as well as humoral immunity in hemodialysis patients and analyze the factors involved in its acquisition. 

This study is particularly important considering additional vaccinations for dialysis patients as diverse VOCs continue to emerge. Since the emergence of the wild strain in 2020, COVID-19 has caused multiple epidemics worldwide. In Japan, the infection spread rapidly after the appearance of the Omicron variant in 2022. Although this variant has a lower rate of severe infection than previous VOCs, it has a higher ability to pass through existing vaccine immunizations and has a higher rate of infection [31]. The importance of increasing antibody titers through a booster vaccination has been established for the healthy population, and a fourth dose of the vaccine has been found to be immunogenic against the Omicron variant and effective in preventing the onset and severity of disease [32]. In contrast, the benefits of the third and subsequent additional vaccinations to dialysis patients have not yet been established, although it is clear that the first and second vaccinations prevented severe illness and reduced mortality in dialysis patients [33]. In addition, new VOCs may emerge and epidemics may occur in future, and continued analysis of the acquisition of humoral and cellular immunity and its capacity to guide the need for additional vaccine administration to hemodialysis patients is a topic for future research.

This study has a few limitations. First, the small cohort size and single-institute study design can cause statistical underpower and may introduce an unknown bias. Second, the detailed characteristics of patients on dialysis, including the duration of dialysis and causative disease, were not included in this study. Third, the survey included questions on previous medical history and medications, which might have contributed to a recall bias. Fourth, the number of days after vaccination at the time of cellular immunity evaluation differed between BNT162b2 and mRNA-1273 vaccinees, rendering simple comparisons difficult. Fifth, the values for the neutralizing activity which were above the detection limit (>500 AU/mL) should be evaluated considering that the exact values are less reliable.

Overall, our research highlights future works as:

(1) To collect a larger number of dialysis cases;
(2) To measure immunity over time; and
(3) To observe the dynamics of immunity after further additional vaccination among dialysis patients.

Addressing these issues will contribute to the understanding of the establishment of post-vaccine immunity in hemodialysis patients more clearly and predict the efficacy of vaccines against SARS-CoV-2 infections.

## 5. Conclusions

This study revealed that an IgG antibody titer against SARS-CoV-2 reached the maximum value two to three weeks after the booster dose among dialysis patients. By repeating the measurements over a period of two months, we clarified the chronological aspects of the acquisition of humoral immunity in dialysis patients after a booster COVID-19 vaccination. In addition, most dialysis patients acquired not only humoral immunity, but also cellular immunity against SARS-CoV-2. These findings suggest that a booster dose vaccination is effective even among dialysis patients. Further research is needed to collect a larger number of cases, to measure immunity over time, and to observe the dynamics of immunity after further additional vaccination among dialysis patients.

## Figures and Tables

**Table 1:** Participant characteristics.

	Total Dialysis Patients (*n* = 58)	3rd Vaccine Type		
		BNT162b2 (*n* = 43)	mRNA-1273 (*n* = 15)	*p*-Value
Age (years, median [IQR])	71 (48–89)	72.7 (51–89)	66 (50–84)	
Females; N (%) *	18 (31.0)			0.03
Interval between 2nd and 3rd vaccinations	232 (198–252)	231 (198–238)	236 (219–252)	
Medical history; N (%)				
Hypertension	53 (91.4)	40 (93.0)	13 (86.7)	0.90
Hyperlipidemia **	7 (12.0)	3 (7.0)	4 (26.7)	<0.01
Bronchial asthma	1 (1.7)	1 (2.3)	0 (0.0)	0.23
Diabetes	27 (48.5)	20 (46.5)	7 (46.7)	0.06
Cardiovascular disease	8 (19.1)	8 (18.6)	0 (0.0)	0.89
Gout **	5 (8.8)	3 (7.0)	2 (13.3)	<0.01
Anaphylaxis	1 (1.7)	1 (2.3)	0 (0.0)	0.23
Respiratory disease	3 (5.2)	2 (4.7)	1 (6.7)	0.58
Rheumatoid arthritis	1 (1.7)	1 (2.3)	0 (0.0)	0.23
Medications; N (%)				
Antihistamine	8 (13.8)	6 (14.0)	2 (13.3)	0.25
NSAIDs	2 (3.4)	2 (4.7)	0 (0.0)	0.23
Steroids	2 (3.4)	2 (4.7)	0 (0.0)	0.23
Acetaminophen **	4 (6.9)	4 (9.3)	0 (0.0)	<0.01
Antitumor agents **	1 (1.7)	0 (0.0)	1 (6.7)	<0.01
Adverse reaction (3rd vaccination); N (%)				
Pain *	9 (15.5)	5 (11.6)	4 (26.7)	0.02
Malaise	5 (8.6)	3 (7.0)	2 (13.3)	0.17
Joint pain **	41 (70.7)	29 (67.4)	12 (80.0)	<0.01
Fever (≥37.5 °C) **	12 (20.7)	6 (14.0)	6 (40.0)	<0.01
Headache	5 (8.6)	4 (9.3)	1 (6.7)	0.50
Fever (<37.5 °C)	6 (10.3)	4 (9.3)	2 (13.3)	0.43
Nausea	1 (1.7)	1 (2.3)	0 (0.0)	0.23

The median (range) or number (percentage) is shown for the continuous or categorical variables. Abbreviations: NSAIDs, non-steroidal anti-inflammatory drugs. The Mann–Whitney U test or Fisher’s exact test was performed. Statistical significance: * *p* < 0.05, ** *p* < 0.01.

**Table 2:** Results of univariate and multivariate analyses of IgG(S) in COVID-19.

	Univariate Analysis		Multivariate Analysis	
	Coef (95% CI)	*p*-Value	Coef (95% CI)	*p*-Value
Age (years) *	−81.8 (−159–4.99)	0.04	−32.02 (−123–58.87)	0.48
Females	939 (−918–2796)	0.32	962 (−905–2829)	0.31
3rd Vaccine type(BNT162b2)	1.48 (−484–3234)	0.14	1000 (−987–2986)	0.32
Medications				
Antihistamine	14.69 (−6.77–36.15)	0.18		
Steroids	−3111 (−7678–1456)	0.18		
Acetaminophen	−125 (−3472–3223)	1.00		
Antitumor agents	1265 (−5236–7765)	0.70		
Medical history				
Hypertension	−1537 (−4858–1784)	0.36		
Hyperlipidemia	395 (−2210–2999)	0.76		
Bronchial asthma	2040 (−4446–8525)	0.53		
Diabetes	862 (−850–2575)	0.32		
Cardiovascular disease	−672 (−3128–1785)	0.59		
Gout	212 (−2810–3235)	0.89		
Anaphylaxis	−1791 (−8283–4699)	0.58		
Respiratory disease	2335 (−1440–6110)	0.22		
Rheumatoid arthritis	−4620 (−11,000–1766)	0.15		
Adverse reaction (3rd vaccination)				
Inoculation site reaction **	3025 (828–5223)	<0.01		
Malaise	2453 (−495–5401)	0.10		
Joint pain, myalgia	1147 (−735–3029)	0.23		
Fever (≥37.5 °C) *	2505 (519–4492)	0.01		
Headache	786 (−2229–3801)	0.60		
Fever (<37.5 °C)	−251 (−3037–2536)	0.86		
Nausea	3990 (−2427–10,400)	0.22		
Presence of systemic adverse effects	1373 (−537–3283)	0.16	886 (−1117–2890)	0.38
Presence of local adverse effects	1657 (−75.57–3389)	0.06	1291 (−577–3159)	0.17

95% CI, 95% confidence interval; aOR adjusted odds ratio, OR odds ratio. Statistical significance: * *p* < 0.05, ** *p* < 0.01.

## Data Availability

Data will be made available on reasonable request from the corresponding author. The data are not publicly available due to privacy or ethical restrictions.

## Supplementary Materials

The following supporting information can be downloaded at: https://www.mdpi.com/article/10.3390/vaccines11020260/s1, Questionnaire S1, S2.
