# Supplementary material for: Antibody and T-Cell Responses against SARS-CoV-2 after Booster Vaccination in Patients on Dialysis: A Prospective Observational Study
Source: Vaccines (Basel). 2023 Jan 25;11(2):260. doi: 10.3390/vaccines11020260 (PMC9962042; doi:10.3390/vaccines11020260)
Supplement: Supplementary file 1 [file vaccines-11-00260-s001.zip › Questionnaire1.pdf]

Japan Agency for Medical Research and Development Vaccine Development Promotion Project

Cohort Study on Vaccination Interval and Antibody Carriage Rate among Medical Care Providers and Elderly People in the Disaster Area of Fukushima Prefecture

COVID-19 antibody test Questionnaire

What can you tell me about vaccines.

Type of Vaccine	Pfizer–BioNTech ・ Moderna ・ Other ( )		
Date of 1st vaccination		Date of 2nd vaccination	

We ask this question to those who have received the second vaccination. Please circle all that apply.

Pain		Fever (<37.5°C)		
Fever (≥37.5°C)		Malaise		
Headache		Joint and muscle pain		
Diarrhea		Nausea		
Dizziness		Other		(symptoms：)

We would like to ask you about your tobacco and alcohol habits. Please circle all that apply.

Currently smoke cigarettes habitually	Yes ・ No
Frequency of drinking alcohol (sake, shochu, beer, Western liquor) and other alcoholic beverages	Daily ・ Occasionally ・ Almost never
Amount of alcohol consumed per day (1 cup of sake = medium bottle of beer = 2 glasses of wine)	Less than 1 cup ・ 1-2 cup ・ 2-3 cup ・ 3 cup or more

Circle "Yes" if you are currently taking any of the following medications on a regular basis, otherwise circle "No".

Steroids (excluding ointments)	Yes ・ No	Drug name: Prednisone, Decadron, Lindelon, etc.
NSAIDs (Antipyretic analgesics)	Yes ・ No	Drug Name:Loxonin,Ibuprofen,Celecox,etc.
Acetaminophen (Antipyretic analgesics)	Yes ・ No	Drug Name:Koronal, etc.
Antihistamines (allergy medications)	Yes ・ No	Drug Name:Allegra,Xyzal,Polaramine etc.
Immunosuppressants	Yes ・ No	(name of a drug ：)
Biologics	Yes ・ No	(name of a drug ：)
Anticancer drugs	Yes ・ No	(name of a drug ：)

If you have any illnesses that you have treated or are currently treating, please circle in the space to the right of the disease name.

COVID-19 (yourself)		COVID-19 (family)	
Hypertension		Gout	
Diabetes		Hyperlipidemia	
Bronchial asthma		Rheumatoid arthritis	
Anaphylaxis		Respiratory disease	
mental disorder		Cardiovascular disease	

	○	name of a disease
Collagen diseases other than rheumatoid arthritis (vasculitis, Behcet disease, scleroderma, SLE, etc.)		
Allergic diseases		
Immunodeficiency diseases (primary congenital immunodeficiency diseases, AIDS, etc.)		
Malignant tumors (hematologic tumors, solid tumors, etc.)		

Thyroid diseases (Graves' disease, hypothyroidism, thyroiditis, thyroid nodule, etc.)		
Liver disease (chronic hepatitis, alcoholic hepatitis, cirrhosis, fatty liver, etc.)		
Kidney disease (IgA nephropathy, dialysis, nephrotic syndrome, etc.)		
Other (please fill in all)		
