# Supplementary material for: Antibody and T-Cell Responses against SARS-CoV-2 after Booster Vaccination in Patients on Dialysis: A Prospective Observational Study
Source: Vaccines (Basel). 2023 Jan 25;11(2):260. doi: 10.3390/vaccines11020260 (PMC9962042; doi:10.3390/vaccines11020260)
Supplement: Supplementary file 1 [file vaccines-11-00260-s001.zip › Questionnaire2.pdf]

Japan Agency for Medical Research Development Vaccine Development Promotion Project

Cohort Study on Vaccination Interval and Antibody Carriage Rate among Medical Care Providers and Elderly People in the Disaster Area of Fukushima Prefecture

COVID-19 antibody test Questionnaire（added after 3rd vaccination）

1. Please answer the following questions about the third vaccination. Please circle all that apply.

Third vaccination	0. vaccinated 1. waiting to be vaccinated 2. not yet vaccinated but will not be vaccinated

2. We ask this question to those who have received the third vaccination. Please circle all that apply.

Type of Vaccine	Pfizer–BioNTech ・ Moderna ・ Other （ ）
Date of 3rd vaccination	

3. We ask this question to those who have already received the third vaccination. Did you have any adverse reactions? Please tick the box to the right of "adverse reactions.

Pain		Fever (<37.5°C)		
Fever (≥37.5°C)		Malaise		
Headache		Joint and muscle pain		
Diarrhea		Nausea		
Dizziness		Other		（symptoms： ）

4. Please tell us if anyone around you has tested positive for COVID-19.

Has anyone in your neighborhood tested positive for COVID-19?	0. No 1. Yes
If you answered "yes," please tell us about your relationship with that person. What is your relationship with this person? (Multiple answers allowed)	0. family members living together 1. non-living family members 2. people at work 3. friends 4. other
If you answered "yes," we would like to ask you the following questions. What kind of contact did you have with that person? (Multiple answers allowed)	1. contact without mask<br>2. contact within 1 meter with or without mask<br>3. contact (talking, eating, drinking, etc.) with or without a mask for more than 15 minutes<br>4. none of the above

5. Please tell us about your new corona infection between December and March.

Whether or not you are infected with COVID-19.	0. No 1. Yes (home treatment) 2. Yes (hospitalization)

If you answered "yes", please answer the following questions. Please circle all that apply to your symptoms. (If you had no symptoms, please leave this box blank.)	Fever (<38.0°C)		Fever (≥38.0°C)	
	Cough		Sore throat	
	Headache		Joint pain, muscle pain	
	Diarrhea		Nausea	
	Malaise		Nasal discharge	
	Other (all listed)			
